# rbcL gene dataset on intra-specific genetic variability and phylogenetic relationship of *Crassocephalum crepidioides* (Benth) S. Moore. (Asteraceae) in Nigeria

**DOI:** 10.1016/j.dib.2023.109266

**Published:** 2023-05-26

**Authors:** Conrad Asotie Omonhinmin, Enameguono Ethel Olomukoro, Chinedu Charles Onuselogu, Jacob Olagbenro Popoola, Stephen Oluwanifise Oyejide

**Affiliations:** a Department of Biological Sciences/Biotechnology Cluster, College of Science and Technology, Covenant University, Ogun State, Nigeria; b Department of Biological Sciences, College of Agriculture, Engineering and Science, Bowen University, PMB 284, 232101, Iwo, Osun State, Nigeria; c Department of Cell Biology and Genetics, Faculty of Science, University of Lagos, Akoka, Nigeria

**Keywords:** *Crassocephalum crepidioide*s, Genetic diversity, Conservation, Evolution, Phylogeny, rbcL gene

## Abstract

*Crassocephalum crepidioides* (Benth) S. Moore (Asteraceae) commonly called “thickhead” is underutilised, indigenous to the rainforest of West and Central Africa but has also been introduced and naturalised throughout tropical and sub-tropical Asia, Australia, Tonga and Samoa. The species is an important medicinal and leafy vegetable endemic to the South-western region of Nigeria. Its cultivation, utilisation and local knowledge base could be stronger than the mainstream vegetables. Genetic diversity is uninvestigated for breeding and conservation purposes. The dataset consists of partial rbcL gene sequences, amino acid profiles and nucleotide compositions for 22 accessions of *C. crepidioides.* The dataset provides information on the species distribution (Nigeria), genetic diversity and evolution. The sequence information is integral for developing specific DNA markers for breeding and conservation purposes.

**Specifications Table**

**Table:** 

Subject	Biological Sciences

Specific subject area	Agricultural, Genetic diversity, Phylogenetics, Evolution.
Type of data	Tables, Figure.
How data were acquired	PCR amplification and DNA Sanger sequencing for rbcL gene amplification.
Data format	Raw, Analyzed.
Description of data collection	Leaf samples of *C. crepidioides* accessions were collected from species endemic areas of Southwest Nigeria, Silica gel dried and preserved under -80°C (Table 1). The population diversity, nucleotide, and amino acid contents of each accession were determined using DnaSP 6.0, and all accessions were assessed using rbcL primers. Phylogenetic tree was constructed using Mega 11.0.13, CodonW was used to estimate the codon use indices and Arlequin 3.5.2.2 used to estimate the AMOVA for the population.
Data source location	The data locations are summarised in Table 1.
Data accessibility	The sequence data of the accessions have been deposited in NCBI GenBank database sequence and has the following accession numbers; MW066441.1, MW066442.1, MW066443.1, MW066444.1 MW066445.1, MW066446.1, MW066447.1, MW066448.1, MW066449.1, MW066450.1, MW066451.1, MW066452.1, MW066453.1, MW066454.1, MW06655.1, MW066456.1, MW066457.1, MW066458.1, MW066459.1, MW066460.1, MW066461.1, MW066462.1 https://www.ncbi.nlm.nih.gov/popset/?term=MW066441.1[1]

## Value of the Data

This dataset is generated for the partial *rbcL* gene sequences and is important for:

• The identification of areas of the abundance of *C. crepidioides* within the study areas is essential for the breeding and germplasm conservation of the species.
• Information on the nucleotide polymorphism, amino acid composition, and Codon usage of *C. crepidioides* accessions collected across South-Western Nigeria.
• The analysis of genetic diversity, molecular phylogeny, evolution and sub-speciation of *C. crepidioides*.
• Information on Codon bias usage, vital for ecological adaptation analysis of the species and the Amino acid profile, essential for the nutritional value and sub-speciation delimitation in the species.

## 1 Objectives of the Study

The objectives were to provide the distribution, intra-specific genetic diversity and phylogenetic relationship of *C. crepidioides* collected across the species endemic areas in Nigeria using rbcL gene sequence.

## 2 Data Description

**Fig. 1:**
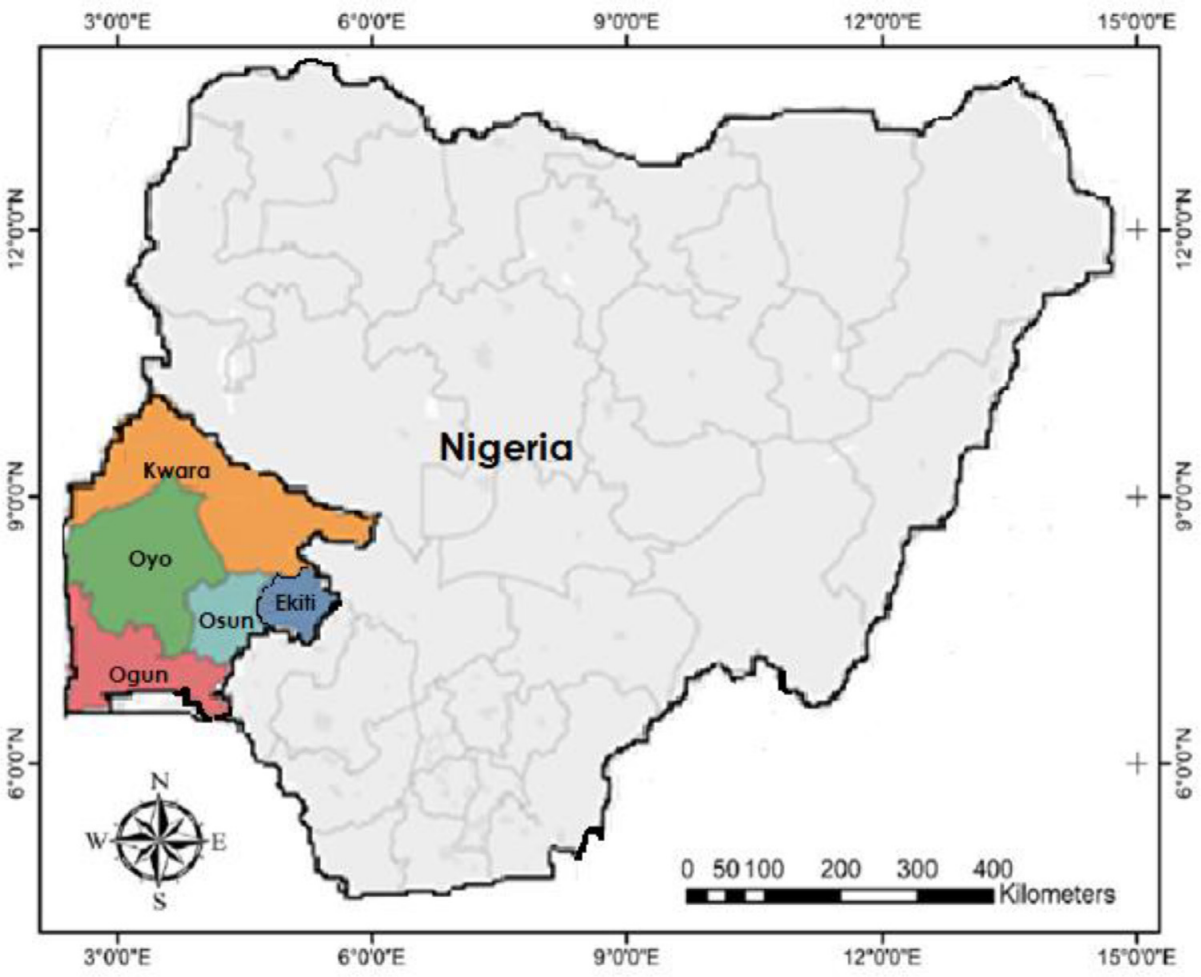
Map showing the study area comprising five states in the South-western region of Nigeria.

**Plate 1:**
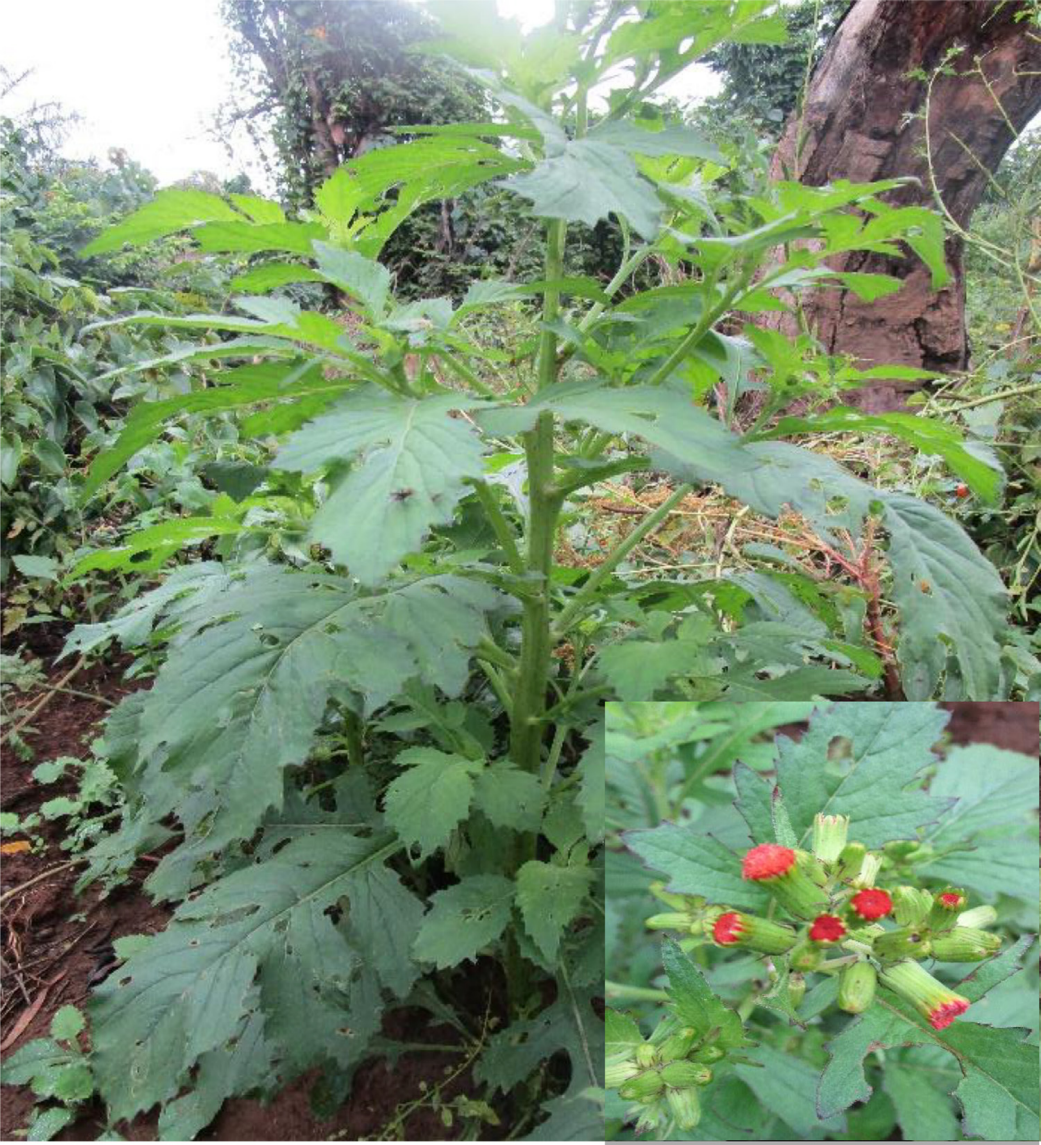
*Crassocephalum crepidioides* plant growing in natural habitat with floral display. (Photo: E.E. Olomukoro)

**Fig. 2:**
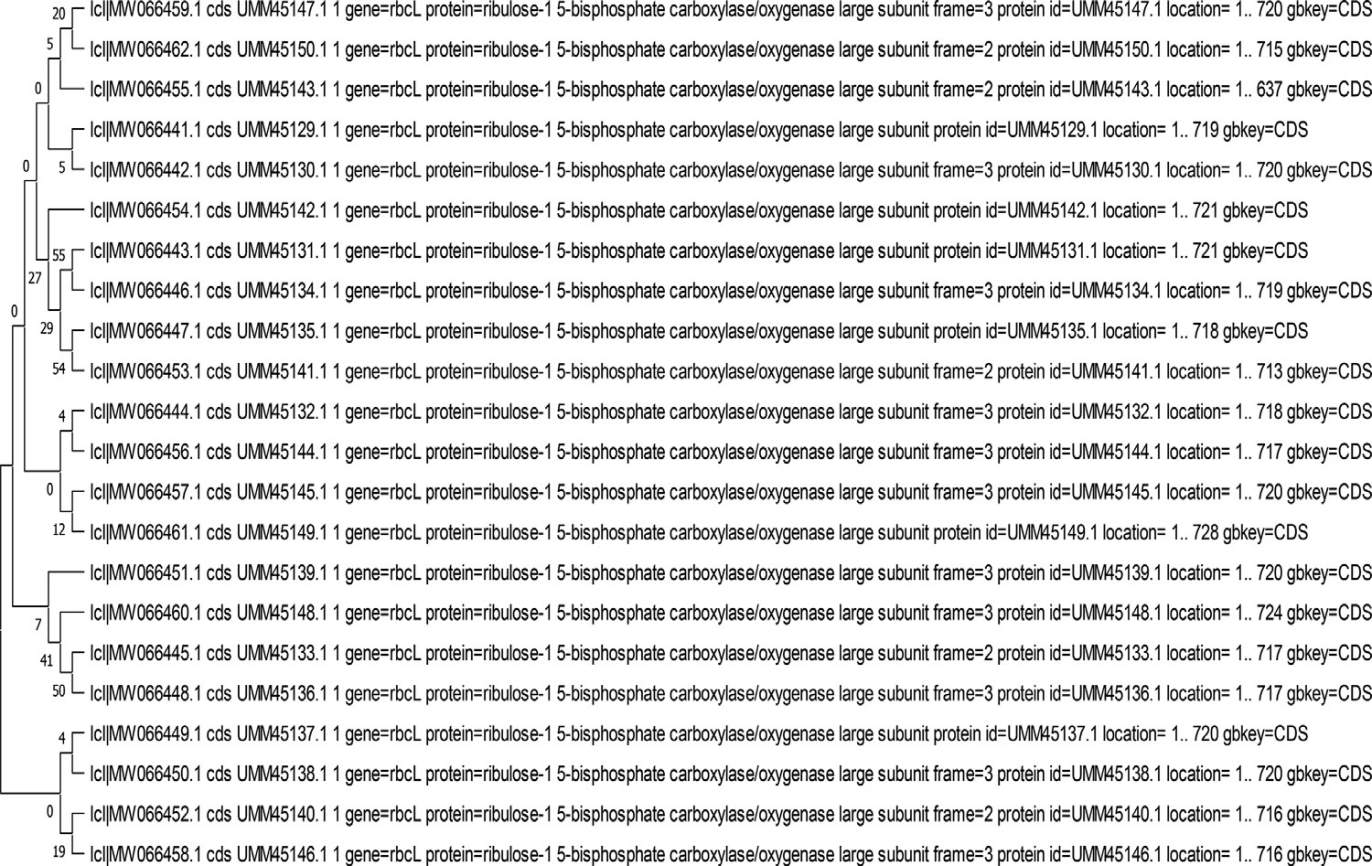
Phylogenetic tree construction using the 22 C. crepidioides rbcL gene sequence information. The low bootstrap reading might be due to high similarity among the sequences.

The present work employs Sanger sequencing for generating sequences of the chloroplast gene - *rbcL* (Ribulose- 1,5-bisphosphate carboxylase/oxygenase large subunit) from 22 accessions of *C. crepidioides* accessions deposited on NBCI GenBank. Fig. 1 shows the collection map for the *C. crepidioides* accessions across five South-western states in Nigeria. Plate 1; shows the *Crassocephalum crepidioides* plant growing in its natural habitat with floral display. Table 1 details the field collection information and the NCBI Genbank accession number for the sequences deposited. Table 2; presents the length of the sequences, % GC and nucleotide content of the 22 *C. crepidioides* sequences. Table 3; records the intra-genetic diversity of the *C. crepidioides* study population. Table 4; presents the genetic diversity information of the species in the study area based on the rbcL sequence analysis. Table 5; represents the codon usage profile based on *C. crepidioides* sequences. Table 6; highlights the Codon usage indices for each of the 22 *C. crepidioides* sequences. Fig. 2; shows the Phylogenetic tree construct using the *C. crepidioides* sequences data. Table 7: records the amino acid molecular weight profile for each of the 22 *C. crepidioides* sequences. Table 8; presents the AMOVA analysis for variance within and between the 5 *C. crepidioides* population.

**Table 1:** Genbank accession number, passport data, location coordinates and altitude of C. crepidioides accessions studied.

SN	Genbank Accession Number (ф)	Herbarium number (voucher)	Area of collection	LGA	State	Latitude NS	Longitude EW	Altitude (m)
1	MW066441.1	ogN001	Agro-Park, Ijale (Farm)	Abeokuta North	Ogun	07°10′20.2′'	3°09′54.1′'	130
2	MW066442.1	ogN002	Ajuba settlement (Farm)	Abeokuta North	Ogun	07°10′16.3′'	3°09′37.4′'	115
3	MW066443.1	oyN002	Omi-Adio (Market)	Ido LGA	Oyo	07°23′35.5′'	3°45′00.9′'	159
4	MW066444.1	oyN003	Agbedo village (Open space)	Akinyele	Oyo	07°34′44′'	3°52′20′'	248
5	MW066445.1	oyN005	Bodija, Ibadan (Market)	Ibadan North	Oyo	07°26′06′'	3°54′52′'	219
6	MW066446.1	oyN006	Bodija, Ibadan (Market)	Ibadan North	Oyo	07°26′06′'	3°54′52′'	222
7	MW066447.1	oyN007	Kosomo, Oyo Road (Open space)	Iseyin LGA	Oyo	07°50′13′'	3°56′39′'	301
8	MW066448.1	oyN009	Oke-Abisa (Farm)	Atisbo LGA	Oyo	8°28′50.8′'	3°26′39.7′'	421
9	MW066449.1	oyN010	Aroje (Farm)	Saki-West	Oyo	08°34′59.3′'	3°23′35.2′'	425
10	MW066450.1	oyN011	Alaro (Farm)	Saki-East	Oyo	08°33′48.2′'	3°29′32.1′'	420
11	MW066451.1	oyN012	Kanga, Kisi Road, (Farm)	Olorunshogo	Oyo	08°45′17.7"	4°07′09.7"	402
12	MW066452.1	oyN013	Igbet-Igboho Road (Open space)	Olorunshogo	Oyo	08°45′19"	4°06’37"	415
13	MW066453.1	oyN014	Orile- Igbon (Farm)	Surulere LGA	Oyo	08°14′31.9′'	4°17′52.5′'	388
14	MW066454.1	oyN015	Ogbomosho-Jebba Road (Farm)	Surulere LGA	Oyo	08°14′30.9′'	4°17′51.0′'	386
15	MW066455.1	kwN001	Taiwo road, near the stadium (Farm)	Ilorin West	Kwara	08°28′31.2′'	4°32′27.2′'	290
16	MW066456.1	kwN002	Oja Oba (Market)	Ilorin West	Kwara	08°29′19′'	4°31′56′'	293
17	MW066457.1	osN001	Oloro, Isundunrin community (Farm)	Ejigbo LGA	Osun	07°52′01′'	4°15′43′'	340
18	MW066458.1	osN002	Ile-saron, Ipetumodu (Open space)	Ife-North	Osun	07°31′35.9′'	4°27′03.1′'	243
19	MW066459.1	ekN004	Ugbo-ugolo, Oye-Ekiti (Farm)	Oye- Ekiti	Ekiti	07°48′14.7′'	5°19′34.0′'	536
20	MW066460.1	ekN005	Ugbo-ugolo, Oye-Ekiti (Farm)	Oye- Ekiti	Ekiti	07°48′12′'	5°19′20′'	550
21	MW066461.1	ekN007	Oja Oba (Market)	Ado-Ekiti	Ekiti	07°37′19′'	5°13′17′'	433
22	MW066462.1	ekN008	Ajowa (Market)	Ado-Ekiti	Ekiti	07°38′02′'	5°13′23′'	436

**Table 2:** Sequence length, percentage nucleotide content and blast search result of the rbcL gene of *C. crepidioides.*

SN	Genbank Accession No.	Sequence Length	%GC	%T	%C	%A	%G
1	MW066441.1	719	42.0	29.5	19.5	28.5	22.5
2	MW066442.1	718	42.1	29.7	19.2	28.3	22.8
3	MW066443.1	721	43.0	29.7	20.2	27.3	22.7
4	MW066444.1	716	41.9	29.9	19.4	28.2	22.5
5	MW066445.1	716	42.6	29.9	19.6	27.5	23.0
6	MW066446.1	717	42.1	29.7	19.2	28.2	22.9
7	MW066447.1	718	41.9	29.5	19.2	28.6	22.7
8	MW066448.1	715	42.1	29.7	19.3	28.3	22.8
9	MW066449.1	720	42.1	29.6	19.2	28.3	22.9
10	MW066450.1	718	42.3	29.7	19.5	28.0	22.8
11	MW066451.1	718	42.3	29.9	19.4	27.7	23.0
12	MW066452.1	715	42.2	30.2	19.6	27.6	22.7
13	MW066453.1	712	43.1	29.1	20.1	27.8	23.0
14	MW066454.1	721	42.3	29.5	19.7	28.2	22.6
15	MW066455.1	636	42.8	29.7	20.0	27.5	22.8
16	MW066456.1	715	41.7	29.9	19.0	28.4	22.7
17	MW066457.1	718	42.1	29.7	19.2	28.3	22.8
18	MW066458.1	714	41.7	30.0	19.2	28.3	22.5
19	MW066459.1	718	42.0	29.7	19.4	28.3	22.7
20	MW066460.1	722	42.1	29.9	19.3	27.7	23.1
21	MW066461.1	728	43.0	29.0	19.4	28.6	23.1
22	MW066462.1	714	41.9	29.7	19.3	27.9	23.1
**Mean**		**714.05**	**42.24**	**29.7**	**19.4**	**28.1**	**22.8**

**Table 3:** Sample size, polymorphic site, haplotype and nucleotide diversity of the 5 Populations of the 22 C. crepidioides accessions studied.

Population	Sample size (n)	Haplotype (h)	Polymorphic sites (S)	Nucleotide diversity (π)	Gene diversity (Hd)
Ekiti	4	4	17	0.013	1.000
Kwara	2	1	0	0.000	0.000
Ogun	2	2	1	0.002	1.000
Osun	2	2	59	0.086	1.000
Oyo	12	12	53	0.017	1.000

**Table 4:** Genetic (Nucleotide) diversity outlay of 22 C. crepidioides accessions based on the rbcL sequence analysis.

Index	Value
Number of haplotypes	15
Haplotype diversity	0.879±0.068
Nucleotide diversity	**0.00611+0.0042**
Average no. Nucleotide Differences (k)	3.870
No. of segregating sites	37
G+C	0.423

**Table 5:** Codon usage bias: Relative Synonymous Codon Usage (RSCU) and count for C. crepidioides rbcL nucleotides.

Codon	Count	RSCU	Codon	Count	RSCU	Codon	Count	RSCU	Codon	Count	RSCU
UUU(F)	8	1.45	UCU(S)	3	2.00	UAU(Y)	10	1.43	UGU(C)	3	1.50
UUC(F)	3	0.55	UCC(S)	3	2.00	UAC(Y)	4	0.57	UGC(C)	1	0.50
UUA(L)	2	0.60	UCA(S)	1	0.67	UAA(*)	0	0.00	UGA(*)	0	0.00
UUG(L)	7	2.10	UCG(S)	0	0.00	UAG(*)	0	0.00	UGG(W)	3	1.00
CUU(L)	5	1.50	CCU(P)	9	2.57	CAU(H)	1	1.00	CGU(R)	7	3.00
CUC(L)	1	0.30	CCC(P)	0	0.00	CAC(H)	1	1.00	CGC(R)	0	0.00
CUA(L)	3	0.90	CCA(P)	3	0.86	CAA(Q)	6	2.00	CGA(R)	3	1.29
CUG(L)	2	0.60	CCG(P)	2	0.57	CAG(Q)	0	0.00	CGG(R)	0	0.00
AUU(I)	4	1.33	ACU(T)	14	2.67	AAU(N)	3	0.75	AGU(S)	1	0.67
AUC(I)	5	1.67	ACC(T)	2	0.38	AAC(N)	5	1.25	AGC(S)	1	0.67
AUA(I)	0	0.00	ACA(T)	4	0.76	AAA(K)	13	1.73	AGA(R)	4	1.71
AUG(M)	2	1.00	ACG(T)	1	0.19	AAG(K)	2	0.27	AGG(R)	0	0.00
GUU(V)	8	2.13	GCU(A)	10	2.00	GAU(D)	10	1.67	GGU(G)	11	2.00
GUC(V)	0	0.00	GCC(A)	4	0.80	GAC(D)	2	0.33	GGC(G)	2	0.36
GUA(V)	6	1.60	GCA(A)	4	0.80	GAA(E)	14	1.56	GGA(G)	6	1.09
GUG(V)	1	0.27	GCG(A)	2	0.40	GAG(E)	4	0.44	GGG(G)	3	0.55

**Table 6:** Codon Usage Indices for each of the 22 C. crepidioides rbcL sequences.

Codon Usage Parameters												
Accessions	T3s	C3s	A3s	G3s	CAI	CBI	Fop	Nc	GC3s	GC	L_sym	L_aa
MW066441.1	0.5487	0.1744	0.3770	0.1379	0.292	0.117	0.491	44.17	0.248	0.418	234	239
MW066442.1	0.5487	0.1744	0.3736	0.1445	0.298	0.130	0.500	43.82	0.252	0.420	234	239
MW066443.1	0.5477	0.1809	0.3799	0.1294	0.288	0.078	0.477	44.02	0.247	0.429	235	240
MW066444.1	0.5487	0.1795	0.3722	0.1404	0.291	0.113	0.489	44.24	0.253	0.417	233	238
MW066445.1	0.5381	0.1777	0.3646	0.1512	0.288	0.104	0.485	44.56	0.262	0.424	233	238
MW066446.1	0.5459	0.1735	0.3757	0.1453	0.292	0.100	0.483	44.10	0.252	0.421	234	239
MW066447.1	0.5408	0.1735	0.3846	0.1387	0.284	0.116	0.491	44.58	0.248	0.418	234	239
MW066448.1	0.5515	0.1753	0.3702	0.1453	0.293	0.110	0.489	44.25	0.253	0.420	233	238
MW066449.1	0.5459	0.1735	0.3716	0.1494	0.287	0.104	0.485	44.47	0.255	0.421	235	240
MW066450.1	0.5482	0.1726	0.3722	0.1462	0.285	0.092	0.479	44.39	0.252	0.423	234	239
MW066451.1	0.5482	0.1777	0.3646	0.1462	0.293	0.108	0.487	43.99	0.256	0.423	234	239
MW066452.1	0.5510	0.1786	0.3743	0.1353	0.301	0.109	0.489	43.40	0.249	0.422	233	238
MW066453.1	0.5385	0.1744	0.3736	0.1445	0.287	0.105	0.487	44.22	0.254	0.430	232	237
MW066454.1	0.5345	0.1839	0.3789	0.1382	0.293	0.114	0.488	43.45	0.256	0.428	207	212
MW066455.1	0.5345	0.1839	0.3789	0.1382	0.293	0.114	0.488	43.45	0.256	0.428	207	212
MW066456.1	0.5515	0.1753	0.3778	0.1404	0.300	0.124	0.498	43.66	0.249	0.416	233	238
MW066457.1	0.5487	0.1744	0.3736	0.1445	0.298	0.130	0.500	43.82	0.252	0.420	234	239
MW066458.1	0.5590	0.1744	0.3743	0.1353	0.298	0.117	0.494	44.00	0.245	0.417	233	238
MW066459.1	0.5459	0.1735	0.3791	0.1387	0.292	0.116	0.491	44.09	0.248	0.420	234	239
MW066460.1	0.5459	0.1735	0.3681	0.1503	0.298	0.120	0.491	44.02	0.256	0.424	234	240
MW066461.1	0.5330	0.1726	0.3777	0.1517	0.289	0.126	0.498	44.93	0.257	0.424	237	242
MW066462.1	0.5459	0.1786	0.3687	0.1471	0.295	0.126	0.498	46.70	0.258	0.424	233	238

**Table 7:** Amino Acid molecular weight profile of the 22 C. crepidioides sequences.

Amino Acid	MW.41.1	MW.42.1	MW. 43.1	MW.44.1	MW.45.1	MW.46.1	MW.47.1	MW.48.1	MW.49.1	MW.50.1	MW.51.1	MW.52.1	MW. 53.1	MW.54.1	MW.55.1	MW.56.1	MW.57.1	MW.58.1	MW.50.1	MW.60.1	MW.61.1	MW.62.1
Ala	8.37	8.79	9.17	8.4	8.82	8.79	8.37	8.82	8.33	9.21	8.79	8.4	10.97	8.33	8.49	8.4	8.79	8.4	8.79	8.33	8.68	8.02
Cys	1.67	1.67	2.08	1.68	1.68	1.67	1.67	1.68	1.67	1.67	1.67	2.1	1.69	1.67	1.89	1.68	1.67	1.68	1.67	1.67	1.65	2.53
Asp	5.02	5.02	5.00	5.04	5.04	5.02	5.02	5.04	5.00	5.44	5.02	5.46	5.06	5.00	5.19	5.04	5.02	5.46	5.02	5.00	4.13	5.06
Glu	7.53	7.53	6.67	7.56	6.72	7.95	7.53	7.98	7.92	7.53	7.95	7.14	7.17	7.5	7.55	7.56	7.53	7.56	7.11	7.92	7.44	7.59
Phe	4.6	5.02	6.25	5.04	5.04	5.02	5.02	5.04	5.00	5.02	5.02	5.46	4.64	5.00	5.19	5.46	5.02	5.46	5.02	5.00	4.55	4.64
Gly	9.21	9.21	10.42	8.82	9.24	9.21	9.21	8.82	9.17	8.79	9.21	9.24	8.86	9.17	9.43	9.24	9.21	9.24	9.21	9.17	9.92	9.7
His	0.84	0.84	0.83	0.84	0.84	0.84	0.84	0.84	0.83	0.84	0.84	0.84	0.84	0.83	0.94	0.84	0.84	0.84	0.84	0.83	0.83	1.27
Ile	3.77	3.77	3.75	3.78	3.78	3.77	3.77	3.78	3.75	3.77	4.18	3.78	3.8	3.75	4.25	3.78	3.77	3.78	3.77	3.75	4.13	3.8
Lys	6.28	6.28	5.00	5.88	5.88	5.44	5.86	5.88	5.83	5.44	5.02	5.46	5.91	5.42	5.19	6.3	6.28	5.88	6.28	5.42	6.2	5.49
Leu	8.37	7.95	6.67	7.98	7.98	7.95	7.95	7.98	7.92	7.95	7.95	7.56	8.02	7.92	8.49	7.56	7.95	7.56	7.95	8.33	7.85	8.02
Met	0.84	0.84	0.83	0.84	0.84	0.84	0.84	0.84	0.83	0.84	0.84	0.84	0.84	0.83	0.94	0.84	0.84	0.84	0.84	1.25	0.83	0.84
Asn	3.35	3.35	3.33	3.78	3.36	3.77	3.35	3.36	3.33	3.77	3.77	2.94	3.38	3.33	3.77	3.36	3.35	3.78	3.35	3.33	3.31	3.8
Pro	5.86	5.86	6.25	5.88	5.88	5.86	5.86	5.88	5.83	5.86	5.86	5.88	5.91	6.25	6.13	5.88	5.86	5.88	5.86	6.25	5.79	5.91
Gln	2.51	2.51	3.33	2.52	2.52	2.51	2.51	2.52	2.5	2.51	2.51	2.94	2.53	2.92	2.83	2.52	2.51	2.52	2.51	2.5	2.89	2.11
Arg	5.86	5.86	5.83	5.88	5.88	5.86	6.28	5.88	6.25	6.28	5.86	5.88	5.91	5.83	6.13	5.88	5.86	5.88	6.28	5.83	5.79	5.91
Ser	3.77	3.77	4.17	3.78	5.04	3.77	3.77	4.2	3.75	3.77	3.77	4.2	4.22	3.75	3.77	3.78	3.77	3.78	3.77	3.75	3.72	3.8
Thr	8.79	8.37	8.33	8.4	7.98	8.37	8.79	8.4	8.75	8.37	8.37	8.4	8.02	9.17	7.55	8.4	8.37	8.4	8.37	7.92	9.09	8.44
Val	6.28	6.28	5.00	6.72	6.3	6.28	6.28	5.88	6.25	5.86	6.28	6.3	5.49	6.25	6.13	6.3	6.28	6.3	6.28	6.67	6.2	6.33
Trp	1.26	1.26	1.25	1.26	1.26	1.26	1.26	1.26	1.25	1.26	1.26	1.26	1.27	1.25	1.42	1.26	1.26	1.26	1.26	1.25	1.24	1.27
Tyr	5.86	5.86	5.83	5.88	5.88	5.86	5.86	5.88	5.83	5.86	5.86	5.88	5.49	5.83	4.72	5.88	5.86	5.46	5.86	5.83	5.79	5.49

**Table 8:** Population differentiation (AMOVA) of the C. crepidioides population studied using the rbcL sequences.

Source of Variation	Sum of Squares	Variance Components	Percentage variation (%)	F-Statistic	p-value
Among Populations	81.273	1.93506	100.00000	F_ST_ = 1.000	<0.05
Within Populations	0.000	0.00000	0.00000		
Total	81.273	1.93506	100		

## 3 Experimental Design, Materials and Methods

### 3.1 Plant Material

Twenty-two (22) *C. crepidioides* accessions were collected from 5 South-Western states in Nigeria, including Oyo, Ekiti, Ogun, Osun and Kwara (Table 1). The plant is indigenous to tropical Africa, particularly in West and Central Africa as a vegetable [2,3]. The leaves samples were carefully cleaned, silica-gel dried, labelled and assigned accession numbers [4]. Herbarium samples were deposited in the repository of the Department of Biological Sciences, Covenant University, Ota Nigeria with assigned voucher numbers. Molecular analysis of the samples was carried out at the Bioscience Laboratory, International Institute of Tropical Agriculture (IITA), Ibadan Nigeria.

### 3.2 Genomic DNA Extraction

Genomic DNA was extracted using the CTAB protocol [5], and quality and quantity were authenticated using the ThermoFischer® Nanodrop spectrophotometer ND-8000-GL

### 3.3 DNA Sequencing and Gene Amplification

The ribulose 1,5 bisphosphate carboxylase/oxygenase large subunit (*rbcL*) gene which served as a reference, was used to design the forward and reverse primers. The sequence of the forward primer (H1f) is 5-ATGTCACCACAAACAGAAAC-3 (T_m_ = 56°C); and the reverse primer (Fofana R): 5-GTAAAATCAAGTCCACCGCG-3 (T_m_ = 56°C) [6,7]. The PCR amplicon was sequenced at the Bioscience Laboratory, International Institute of Tropical Agriculture (IITA) Ibadan, Nigeria using the ABI 3130x genetic analyser (Applied Biosystems)

### 3.4 Data Analysis

The Sanger sequences generated were aligned using ClustalW on BioEdit (ver. 7.2.5) with default settings to create a consensus sequence [8]. Sequences were submitted on NCBI GenBank with accession numbers in Table 1. Population diversity indices such as numbers of segregating sites (S), haplotype number (h), haplotype diversity (Hd), and nucleotide diversity (π) and the codon usage frequency table of *C. crepidioides* were estimated using DnaSP 6.0 [9]. The nucleotide composition, amino acid compositions and phylogenetic analyses were conducted in MEGA v11.0.13 [10]. Codon usage indices were calculated using CodonW as implemented on a public Galaxy server (https://galaxy.pasteur.fr/). Arlequin 3.5.2.2 was used to estimate the Analysis of Molecular Variance (AMOVA) within the populations [11].

## Ethics Statements

The field data presented in Table 1 were obtained via open field collection visits and did not require informed consent. No part of the data was obtained from any Social Media platform.

## CRediT Author Statement

**Conrad Asotie Omonhinmin**: Secure Funding, Conceptualization, Methodology, Supervision; Review manuscript; **Enameguono Ethel Olomukoro**: Experimentation, Writing manuscript, Submission of Sequences on GenBank; **Jacob Olagbenro Popoola**: Writing manuscript, Methodology, review of manuscript; **Chinedu Charles Onuselogu**: Data analysis, writing – original draft preparation, review, and editing manuscript; **Stephen Oluwanifise Oyejide:** Data analysis, Review and Editing of the manuscript.

## Declaration of Competing Interest

The authors declare that they have no known competing financial interests or personal relationships that could have appeared to influence the work reported in this paper.

## Data Availability

RbcL Sequence Diversity in Crassocephalum crepidioides (Benth) S. Moore from Five Selected States in Nigeria (Original data) (GenBank NCBI).
